# Deep top-down proteomics revealed significant proteoform-level differences between metastatic and nonmetastatic colorectal cancer cells

**DOI:** 10.1126/sciadv.abq6348

**Published:** 2022-12-21

**Authors:** Elijah N. McCool, Tian Xu, Wenrong Chen, Nicole C. Beller, Scott M. Nolan, Amanda B. Hummon, Xiaowen Liu, Liangliang Sun

**Affiliations:** ^1^ Department of Chemistry, Michigan State University, 578 S Shaw Lane, East Lansing, MI 48824, USA.; ^2^ Department of BioHealth Informatics, Indiana University–Purdue University Indianapolis, 719 Indiana Avenue, Indianapolis, IN 46202, USA.; ^3^ Department of Chemistry and Biochemistry, The Ohio State University, 100 West 18th Avenue, Columbus, OH 43210, USA.; ^4^ The Comprehensive Cancer Center, The Ohio State University, 500 West 12th Avenue, Columbus, OH 43210, USA.; ^5^ Deming Department of Medicine, School of Medicine, Tulane University, 1441 Canal Street, New Orleans, LA 70112, USA.

## Abstract

Understanding cancer metastasis at the proteoform level is crucial for discovering previously unknown protein biomarkers for cancer diagnosis and drug development. We present the first top-down proteomics (TDP) study of a pair of isogenic human nonmetastatic and metastatic colorectal cancer (CRC) cell lines (SW480 and SW620). We identified 23,622 proteoforms of 2332 proteins from the two cell lines, representing nearly fivefold improvement in the number of proteoform identifications (IDs) compared to previous TDP datasets of human cancer cells. We revealed substantial differences between the SW480 and SW620 cell lines regarding proteoform and single amino acid variant (SAAV) profiles. Quantitative TDP unveiled differentially expressed proteoforms between the two cell lines, and the corresponding genes had diversified functions and were closely related to cancer. Our study represents a pivotal advance in TDP toward the characterization of human proteome in a proteoform-specific manner, which will transform basic and translational biomedical research.

## Teaser

Top-down proteomics of colorectal cancer cells provides proteoform-level knowledge about cancer metastasis.

## INTRODUCTION

Colorectal cancer (CRC) is the third most common cancer worldwide and has a high mortality rate even with recent improvements in therapies (*1*, *2*). CRC metastasis is the main cause of CRC-related death. New insights into the molecular mechanisms of CRC metastasis will undoubtedly be beneficial for developing more effective drugs (*3*–*5*). Extensive studies have been completed with the goal of understanding CRC metastasis at the transcriptome level, generating tremendous information about the landscape of mRNA across different stages of CRC (*6*, *7*). However, nucleic acid–based measurements do not correlate well with protein abundance, which are the primary effectors of function in biology (*8*). Quantitative bottom-up proteomics (BUP) studies of metastatic and nonmetastatic CRC cell lines have found previously unidentified protein regulators involved in CRC metastasis (*4*, *9*, *10*). BUP usually provides limited information on the proteoforms, which represent all possible protein molecules derived from the same gene resulting from genetic variations, RNA alternative splicing, and protein posttranslational modifications (PTMs) (*11*, *12*). Mass spectrometry (MS)–based top-down proteomics (TDP) directly measures intact proteoforms and provides opportunities to study functions of specific proteoforms (*13*, *14*). Unfortunately, there is still no report in the literature about studying CRC metastasis using TDP, and this study will help to fill that gap.

**Fig. 1.**
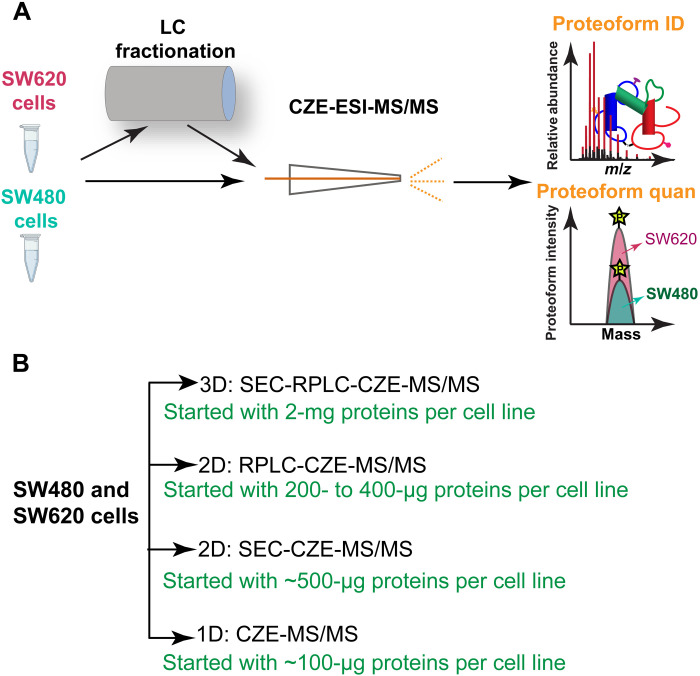
Schematic of the experimental design. (**A**) Schematic design of the TDP study of metastatic (SW620) and nonmetastatic (SW480) CRC cells using CZE-ESI-MS/MS and LC-CZE-ESI-MS/MS for proteoform ID and LFQ. (**B**) Four CZE-MS/MS–based strategies in this work with the amounts of protein starting materials.

Here, we performed the first deep TDP study of metastatic (SW620) and nonmetastatic (SW480) human CRC cell lines, aiming to produce a comprehensive proteoform-level view of the two isogenic CRC cell lines and discover novel proteoform biomarkers of CRC metastasis. We used four different capillary zone electrophoresis (CZE)–tandem MS (MS/MS) approaches, one-dimensional (1D) CZE-MS/MS, 2D size exclusion chromatography (SEC)–CZE-MS/MS, 2D reversed-phase liquid chromatography (RPLC)–CZE-MS/MS, and 3D SEC-RPLC-CZE-MS/MS analyses of the two cell lines for proteoform identification (ID) and label-free quantification (LFQ) (Fig. 1). For 1D CZE-MS/MS, each sample was analyzed by CZE-MS/MS in technical triplicate. For 2D SEC-CZE-MS/MS, each sample was fractionated by SEC into six fractions, followed by CZE-MS/MS in technical triplicate. For 2D RPLC-CZE-MS/MS, we fractionated each sample to 6 or 13 fractions by RPLC and analyzed each LC fraction by single-shot CZE-MS/MS (RPLC 13 fractions) or triplicate CZE-MS/MS measurements (RPLC 6 fractions). For 3D SEC-RPLC-CZE-MS/MS, 52 LC fractions were collected for each sample, followed by CZE-MS/MS in technical triplicate. From 1D separation to 3D separations, the required amount of starting protein materials increased (from 100 μg to 2 mg) because of the unavoidable sample loss during sample collections and transfers. The TopPIC (version 1.4.0) software was used for data analysis (*15*), and a 1% proteoform-level false discovery rate (FDR) was used to filter the database search results.

## RESULTS

### ID of more than 23,000 proteoforms from CRC cells using CZE-MS/MS

One long-term goal of TDP is to characterize all the millions of proteoforms in the human body (*16*, *17*). During the past decade, because of the improvement of proteoform sample preparation, LC and CZE separations, MS, and MS/MS, 3000 to 5000 proteoforms corresponding to roughly 1000 genes can be identified from one human cell line using LC-MS/MS–based platforms (*18*–*22*), and up to 6000 proteoform IDs corresponding to 850 genes have been reported from an *Escherichia coli* sample using a CZE-MS/MS–based workflow (*23*). Only one TDP study of a human cell line using CZE-MS/MS was reported with the ID of about 500 proteoforms (*24*). Recently, the Kelleher group (*21*) reported the ID of ~30,000 proteoforms of 1690 human genes from 21 human cell types and plasma using RPLC-MS/MS–based strategies, representing a milestone in large-scale TDP. On average, nearly 3000 proteoforms were identified from one of the 21 human cell types.

**Fig. 2.**
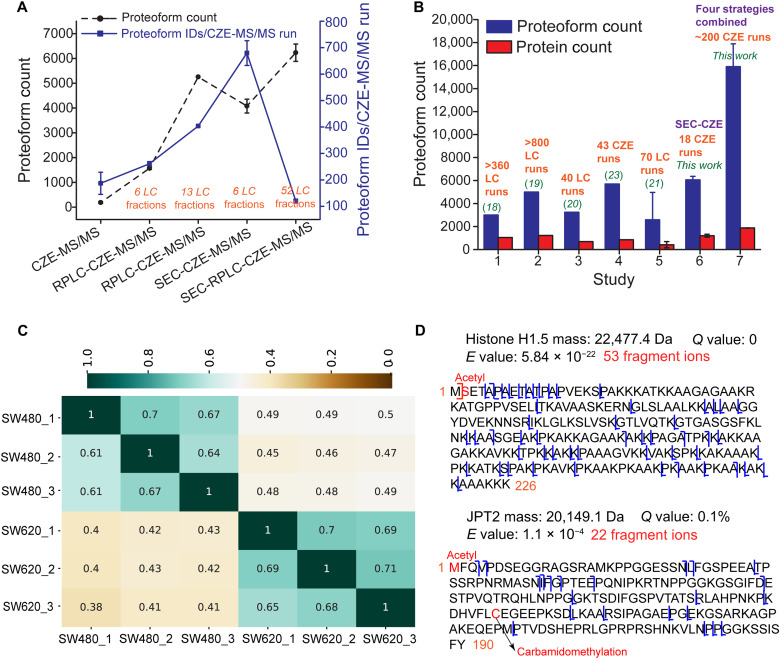
Summary of proteoform ID results of this study. (**A**) Proteoform IDs from SW480 cells using different CZE-MS/MS–based strategies. The error bars represent the SDs of the number of proteoform IDs from technical triplicates. (**B**) The number of proteoform and protein IDs per complex proteome sample using RPLC- or CZE-MS/MS–based TDP strategies. The data of studies 5 to 7 are shown as means ± SDs from various proteome samples. For example, the mean and SDs of proteoform and protein counts from SW480 and SW620 cells are shown in studies 6 and 7. (**C**) Heatmap of proteoform overlaps from technical triplicates of SW480 and SW620 cells using SEC-CZE-MS/MS. Each number in the figure represents a ratio between the number of shared proteoforms in two conditions [e.g., SW480_1 (*x* axis) and SW620_1 (*y* axis)] and the total number of identified proteoforms in one of the two conditions listed on the *y* axis (e.g., SW620_1). For example, the proteoform overlap between SW480_1 (*x* axis) and SW620_1 (*y* axis) is 0.4, which indicates the ratio between the number of shared proteoforms in those two conditions and the total number of identified proteoforms in SW620_1. (**D**) Sequences and fragmentation patterns of identified example proteoforms in the study.

In this work, we performed the first global TDP study of a pair of isogenic human nonmetastatic and metastatic CRC cell lines (SW480 and SW620). Four different strategies were used Fig. 1. We first compared the four different CZE-MS/MS strategies listed in Fig. 1B in terms of the number and efficiency of proteoform IDs from the SW480 cells Fig. 2A. SEC-RPLC-CZE-MS/MS outperformed SEC-CZE-MS/MS, RPLC-CZE-MS/MS, and CZE-MS/MS in terms of the number of proteoform IDs due to better LC fractionation (2D LC versus 1D or no LC) and much more CZE-MS/MS runs (52 versus 6 and 13). In terms of the proteoform ID efficiency (the number of proteoform IDs per CZE-MS/MS run), the SEC-CZE-MS/MS (six LC fractions) produced nearly 700 proteoform IDs per run, which is nearly six- and fourfold higher than those from SEC-RPLC-CZE-MS/MS and CZE-MS/MS, respectively. We drew two conclusions from the data. First, multidimensional separation is crucial for large-scale TDP analysis of human cell lysates due to their extremely high complexity. Second, SEC-CZE-MS/MS and RPLC-CZE-MS/MS under an optimized condition are powerful techniques for deep TDP of human cell lysates with high throughput.

In total, we collected more than 400 MS raw files using the four CZE-MS/MS–based strategies and identified 23,622 proteoforms of 2332 proteins from the SW480 and SW620 cell lines with a 1% proteoform-level FDR. The number of proteoform IDs from the CRC cells is about five- to eightfold higher than that reported in previous TDP studies of human cancer cells (23,622 versus 3000 to 5000 proteoforms) (*18*–*20*). A total of 17,316 and 14,504 proteoforms (on average 15,910 proteoforms) were identified from SW480 and SW620 cell lines, respectively, representing about threefold improvement in the number of proteoform IDs per human cell line compared to previous LC-MS/MS–based TDP datasets. The number of proteoform IDs is about 30-fold higher than previous human cell TDP datasets by CZE-MS/MS (~16,000 versus ~500) (*24*). Figure 2B shows the number of proteoform IDs per complex sample using TDP in previous works and this study (*18*–*23*). Table S1 summarizes the details of those studies.

We need to point out that the nearly 16,000 proteoform IDs from SW480 or SW620 cells combine the results of four different CZE-MS/MS–based strategies and about 200 CZE-MS/MS runs. The previous literature studies typically use one LC-MS/MS or CZE-MS/MS–based approach (*18*–*23*). We also included the data of SW480 and SW620 cells from only SEC-CZE-MS/MS in Fig. 2B. A total of 5855 and 6273 proteoforms (mean ± SD: 6064 ± 296) were identified from SW480 and SW620 cells, respectively, by SEC-CZE-MS/MS, via 18 CZE-MS/MS runs (6 SEC fractions × 3 CZE-MS/MS runs/fraction). The SEC-CZE-MS/MS produced much higher proteoform IDs (6000 versus 3000 to 5000) from a single human cell line than LC-MS/MS–based approaches in the literature with a drastically lower number of MS runs (18 versus 40 to 800).

The data clearly demonstrate the power of our CZE-MS/MS–based TDP strategy for comprehensive characterization of proteoforms in complex proteome samples. We attribute the drastic improvement of proteoform IDs to the high separation efficiency of CZE for proteoforms (*25*), high sensitivity of CZE-MS for proteoform detection (*25*–*27*), and high orthogonality of LC and CZE for biomolecule separations (*23*, *28*). The features of CZE-MS/MS for TDP have been systematically reviewed recently (*29*, *30*). The list of identified proteoforms is shown in data file S1.

We further compared the proteoforms and proteins identified from the SW480 and SW620 cells using the SEC-CZE-MS/MS data. Figure 2C shows that the heatmap of proteoform overlaps among technical replicates of SW480 and SW620 cells. About 60 to 70% of proteoforms identified in one technical replicate of SW480 or SW620 cells were also identified in another replicate of the same cell line, indicating reasonable reproducibility of proteoform ID using SEC-CZE-MS/MS and the data-dependent acquisition mode. Figure S1 shows base peak electropherograms of triplicate CZE-MS/MS measurements of the SW620 cell lysate (one SEC fraction), indicating good reproducibility of CZE-MS/MS for complex proteome samples regarding separation profile and base peak intensity. Only about 40 to 50% of proteoforms identified in one replicate of SW480 cells (e.g., SW480_1) were identified in one replicate of SW620 cells (e.g., SW620_1). The proteoform overlaps in Fig. 2C between the two cell lines are statistically significantly lower than that within each cell line (44 ± 4% versus 67 ± 4%, *P* < 10^−14^, two-tailed Student’s *t* test). The data clearly demonstrate that the pair of isogenic human nonmetastatic (SW480) and metastatic (SW620) CRC cell lines have substantially different proteoform profiles. The two cell lines are also different at the protein level, as demonstrated by the protein overlaps shown in fig. S2. The difference in protein overlaps between the two cell lines and within each cell line is statistically significant (69 ± 8% versus 83 ± 3%, *P* < 10^−6^, two-tailed Student’s *t* test).

TDP has some technical challenges for the ID of large proteoforms (i.e., >30 kDa). In this work, we focused on the characterization of proteoforms smaller than 30 kDa using a Thermo Q-Exactive HF mass spectrometer. Figure S3 shows the mass distribution of identified proteoforms from SW480 and SW620 cells. The majority of identified proteoforms are 10 kDa or smaller, which is one main limitation of this study. It is worth noting that 1600 to 2200 proteoforms have masses larger than 10 kDa. Figure 2D shows the sequences and fragmentation patterns of two example proteoforms. Those two proteoforms were identified with high confidence and were also well characterized with N-terminal methionine removal and N-terminal acetylation.

### Proteoforms of important genes in well-known CRC-related pathways

**Fig. 3.**
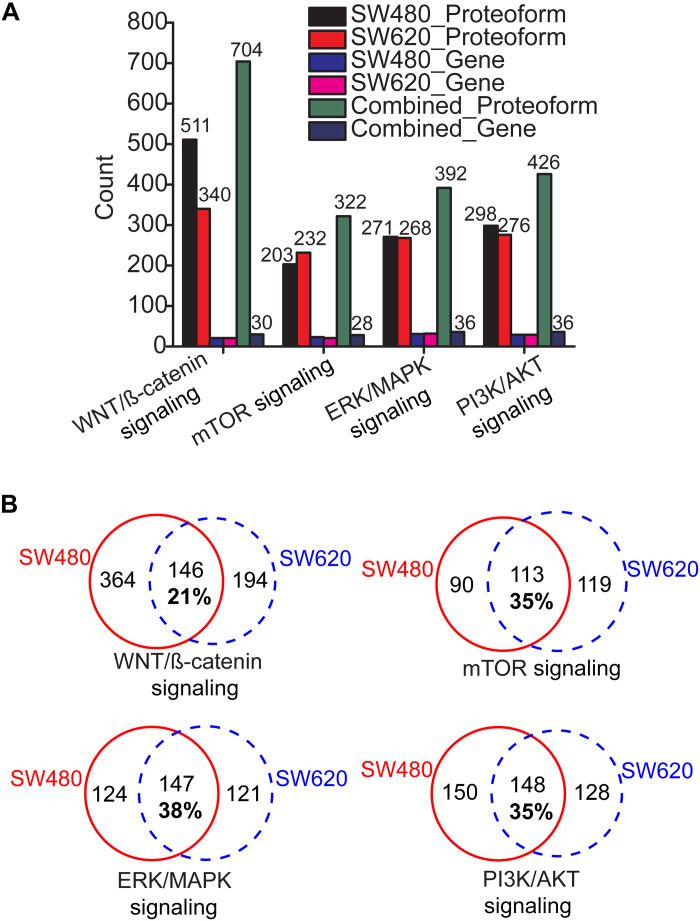
Summary of proteoforms from genes involved in well-known CRC-related pathways. (**A**) The number of proteoforms and genes in four CRC-related pathways identified from SW480 and SW620 cells. (**B**) Overlaps of identified and pathway-related proteoforms between SW480 and SW620 cells.

We further performed QIAGEN Ingenuity Pathway Analysis (IPA) analysis of the genes identified in this work by the four CZE-MS/MS–based strategies and determined several significantly enriched and well-known CRC-related pathways, including WNT/β-catenin signaling (*P* value: 10^−3^), phosphoinositide-3-kinase (PI3K)/Protein kinase B (Akt) signaling (*P* value: 10^−4^), mammalian target of rapamycin (mTOR) signaling (*P* value: 10^−14^), and extracellular signal–regulated kinase (ERK)/mitogen-activated protein kinase (MAPK) signaling pathways (*P* value: 10^−4^) (*31*, *32*). Those pathways play critical roles in CRC progression via regulating cell proliferation, apoptosis, survival, etc. We identified hundreds of proteoforms from dozens of genes for each pathway (Fig. 3A). The lists of proteoforms are shown in data file S1. Comparable numbers of proteoforms were identified from SW480 and SW620 cells for PI3K/AKT signaling, mTOR signaling, and ERK/MAPK signaling pathways. An obviously higher number of proteoforms was obtained from SW480 cells compared to SW620 cells for the WNT/β-catenin signaling pathway (511 versus 340). Combination of the data from SW480 and SW620 cells produced about 40% more proteoforms related to the four CRC pathways compared to one cell line alone, indicating the potential differences in proteoform profiles for the well-known CRC-related pathways between the nonmetastatic and metastatic CRC cell lines. As shown in Fig. 3B, the shared proteoforms between SW480 and SW620 cells for each pathway is only about 21 to 38% of the total proteoforms identified from the two cell lines. The data suggest that proteoforms in those pathways could potentially play important roles in driving CRC progression and metastasis.

We highlighted some proteoforms of important genes (*MARK2*, *SOX9*, *EIF4B*, and *EIF4EBP1*) related to the WNT/β-catenin signaling, mTOR signaling, and PI3K/AKT signaling pathways in Table 1. *MARK2* plays vital roles in modulating directional cancer cell migration, which is crucial for cancer metastasis (*33*). SRY-Box Transcription Factor 9 (*SOX9*) is a high-mobility group box transcription factor and plays essential roles in regulating CRC progression (*34*). Expression of *SOX9* is closely associated with the 5-year overall survival rate of patients with CRC (*34*). Eukaryotic Translation Initiation Factor 4B (*EIF4B*) regulates cancer cell proliferation and has been reported as a potential target for developing anticancer therapies (*35*). Phosphorylation of Eukaryotic Translation Initiation Factor 4E Binding Protein 1 (*EIF4EBP1*) has been reported as an important regulator of cancer progression (*36*).

**Table 1.** Summary of selected proteoforms of important genes. Those genes are related to WNT/β-catenin signaling, mTOR signaling, and PI3K/AKT signaling pathways. “×” suggests that the proteoform is identified in the sample. “ND” indicates that the proteoform is not identified in the sample.

Gene	Pathway	Proteoform	SW480	SW620	Proteoform intensities (SW480/SW620)*
*MARK2*	WNT/β-catenin signaling	M.(S)[acetyl]SARTPLPTLNERDTEQPTLGHLDSK(PSSKSNMIRGRNSAT) (mass shift: 96 Da, phospho and oxidation)SADEQPHIGNY.R	×	ND	4.8 × 10^5^/2.8 × 10^4^
*SOX9*	WNT/β-catenin signaling	R.SQYDYTDHQNSSSYYSHAAGQGTGLYSTFTYMNPAQRPMYTPIADTSGV(PSIPQTHS) (mass shift: 78 Da, phospho)PQHWEQPVYTQLTRP.	×	ND	3.0 × 10^5^/4.6 × 10^4^
*EIF4B*	mTOR signaling	M.AASAKKKNK(KGKTISLTDFL) (mass shift: 122 Da, phospho and acetylation/trimethylation)AEDGGTGGGSTYVSKPVSWADETDDLEGDVSTTWHSNDDDVYRAPPIDRSILPTAPR.A	ND	×	7.5 × 10^4^/4.4 × 10^5^
*EIF4B*	mTOR signaling	M.(A)[acetyl]ASAKKKNKKGKTISLTDFLAEDGG(T) (mass shift: 80 Da, phospho)GGGSTYVSKPVSWADETDDLEGDVSTTWHSNDDDVYRAPPIDR.S	ND	×	5.0 × 10^4^/3.1 × 10^5^
*EIF4EBP1*	PI3K/AKT signaling	.MSGGSS(C)[carbamidomethylation]SQTPSRAIPAT(RRVVLGDGVQLPPGDYSTT) (mass shift: 81 Da, phospho) PGGTLFSTTPGGTRIIYDRKFLME(C) (carbamidomethylation) RNSPVTKTPPRDLPTIPGVTSPSSDEPPMEASQSHLRNSPEDKRAGGEESQFEMDI	ND	×	6.0 × 10^4^/3.5 × 10^6^
*EIF4EBP1*	PI3K/AKT signaling	K.TPPRDLPTIPGVTS(PSSDEPPMEASQSHLRNS) (mass shift: 81 Da, phospho) PEDKRAGGEESQFEMDI	×	ND	1.5 × 10^5^/5.0 × 10^4^

*The proteoform intensities were observed by manually checking the raw data based on the migration time, charge, and *m*/*z* of proteoforms in the database search results. The average intensity of the identified charge state of each proteoform across the proteoform peak is shown in the table.

We identified some phosphorylated proteoforms of those genes, which are unique to either SW480 or SW620 cells (Table 1). For example, two phosphorylated proteoforms of *MARK2* and *Sox9* in the WNT/β-catenin signaling were exclusively identified in the SW480 cells; two phosphorylated proteoforms of *EIF4B* in the mTOR signaling pathway were identified solely in the SW620 cells. SW480 and SW620 cells have different phosphorylated proteoforms of *EIF4EBP1* in the PI3K/AKT signaling pathway. We further manually checked the intensities of those proteoforms in the SW480 and SW620 raw files by matching the mass/charge ratio (*m*/*z*), charge state, and migration time information from the database search. The proteoform intensity data agree well with the database search results (Table 1). For example, the three phosphorylated proteoforms identified solely in SW620 cells have roughly 6- to 60-fold higher intensity in SW620 cells compared to SW480 cells. The extracted ion electropherograms (EIEs) of the two *EIF4B* phosphorylated proteoforms from triplicate CZE-MS/MS analyses are shown in figs. S4 and S5. The data further suggests good reproducibility of proteoform measurements in terms of base peak proteoform intensity from technical triplicates (relative SDs of ≤25%). Protein phosphorylation is well known for modulating cancer progression, including CRC. Although the roles of those four genes in regulating cancer progression have been well studied, the specific functions of those phosphorylated proteoforms of the genes have not been investigated. Here, we documented the remarkable differences in protein phosphorylation of those genes between nonmetastatic and metastatic CRC cell lines in a proteoform-specific manner. Those phosphorylated proteoforms could be central to the progression of CRC metastasis.

### Proteoforms with PTMs and single amino acid variants

Protein PTMs modulate their biological function. For example, protein N-terminal acetylation influences the stability, folding, binding, and subcellular targeting of proteins (*37*). Protein phosphorylation is well known for regulating cell signaling, gene expression, and differentiation (*38*). Protein methylation plays important roles in modulating transcription (*39*). All the data analyses in the following parts of the manuscript are based on the combined data from SEC-CZE-MS/MS, RPLC-CZE-MS/MS, and SEC-RPLC-CZE-MS/MS corresponding to 23,319 proteoforms (data file S1) unless specified otherwise.

**Fig. 4.**
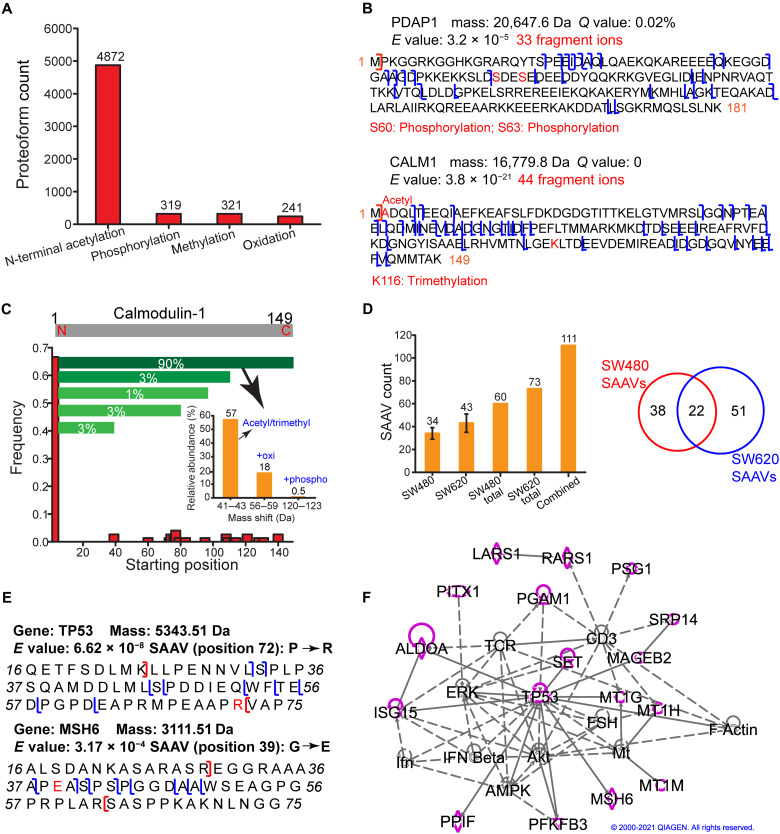
Analyses of the identified proteoforms from CRC cells with PTMs and SAAVs. (**A**) Proteoforms with various PTMs, including N-terminal acetylation, phosphorylation, methylation, and oxidation. (**B**) Sequences and fragmentation patterns of two proteoforms, one proteoform of PDAP1 with two phosphorylation sites and one proteoform of CALM1 with N-terminal acetylation and one lysine trimethylation. (**C**) Summary of all the identified proteoforms of CALM1 regarding starting positions, relative abundance based on the number of PrSMs, and PTMs. (**D**) The number of proteoforms containing SAAVs identified from the SW480 and SW620 cells and the overlap of those proteoforms. The SEC-CZE-MS/MS and RPLC-CZE-MS/MS (RPLC 6 fractions) data were used for the analysis. The error bars in the figure represent the SDs of proteoforms from triplicate measurements. (**E**) Sequences and fragmentation patterns of two proteoforms containing SAAVs. (**F**) SAAVs containing proteoforms correspond to many genes (highlighted in purple) that are involved in a cancer-related network according to the IPA analysis. The diamond, triangle, oval, and circle shapes represent proteins belonging to enzyme, phosphatase/kinase, transcription regulator, and others, respectively. The solid and dotted lines represent direct and indirect interactions. Copyright permission has been granted by QIAGEN for using the network data.

This large-scale TDP study identified 4872 proteoforms with N-terminal acetylation (+42-Da mass shift), 319 proteoforms with phosphorylation [+80-Da (single phosphorylation) or +160-Da (double phosphorylation) mass shift], 321 proteoforms with methylation (+14-Da mass shift), and 241 proteoforms with oxidation (+16-Da mass shift) (Fig. 4A). TDP is powerful for the characterization of combinations of various PTMs on proteoforms. Here, we identified 54 proteoforms with two phosphorylation sites and 90 proteoforms with both acetylation and phosphorylation PTMs. Figure 4B shows the sequences and fragmentation patterns of 28-kDa heat- and acid-stable phosphoprotein (PDAP1) and calmodulin-1 (CALM1) proteoforms with either two phosphorylation sites or the combination of N-terminal acetylation and one lysine trimethylation. Those PTMs of the two proteins agree with the literature data (*40*, *41*). Those two proteoforms were identified with high confidence and were well characterized in terms of PTMs. PDAP1 and CALM1 are both prognostic markers of cancer according to the Human Protein Atlas (www.proteinatlas.org/). However, the potential roles of those specific proteoforms of PDAP1 and CALM1 in cancer are still not clear. The capability of TDP for delineating those proteoforms opens the door of further investigating their potential functions in CRC.

One important value of TDP is its capability for delineation of various proteoforms from the same gene (proteoform family) (*42*). Figure 4C shows one example of *CALM1* proteoform family. CALM1 modulates many enzymes (kinases and phosphatases), ion channels, and many other proteins by calcium binding. We identified 75 proteoforms of *CALM1*. Nearly 70% of those proteoforms start at the position 2 with the N-terminal methionine removal. Various truncated proteoforms, for example, with the starting positions around 40, 60, 80, and 120, were identified in a much lower frequency. The number of proteoform spectrum matches (PrSMs) can be used to roughly estimate the relative abundance of proteoforms (*21*). For the *CALM1* proteoforms starting from position 2, about 90% of the corresponding PrSMs match to proteoforms covering the whole protein sequence (2 to 149), called intact proteoforms. The PrSMs corresponding to other C-terminally truncated proteoforms only account for 3% or lower. The intact proteoforms have various PTMs, including acetylation/trimethylation, oxidation, and phosphorylation. The intact proteoforms of *CALM1* with a 42-Da mass shift (acetylation/trimethylation) are the most abundant forms; intact proteoforms with additional oxidation (a 58-Da mass shift) or phosphorylation (a 122-Da mass shift) have much lower abundance according to the number of PrSMs of those proteoforms.

Cancers result from gene mutations, which produce proteoforms containing amino acid variants (AAVs). Although transcriptomic analysis can provide ample information about gene mutations and possible AAVs on proteins, it is valuable to detect proteoforms containing AAVs directly because gene expression can be regulated posttranscriptionally. BUP has been used for the ID of peptides containing single AAVs (SAAVs) from cancer cells (*43*). The Kelleher group (*44*) reported the ID of 10 proteoforms containing SAAVs from breast tumor xenografts in one TDP study. Here, we identified 111 proteoforms containing SAAVs of 82 genes from the SW480 and SW620 cell lines with a proteogenomic approach with a 5% proteoform-level FDR, representing one order of magnitude improvement in the number of identified proteoforms containing SAAVs compared to previous studies of cancer cells (Fig. 4D). The SEC-CZE-MS/MS and RPLC-CZE-MS/MS (RPLC 6 fractions) data were used for the analysis. The transcriptomic variants based on the available RNA sequencing (RNA-seq) data were incorporated into the protein database for the ID of proteoforms containing SAAVs using TopPG, a recently developed bioinformatics tool (*45*). We also manually inspected the MS/MS spectra of proteoforms containing the SAAV sites to ensure high-confidence IDs. Only 20% of the 111 proteoforms were identified from both cell lines, indicating potentially different SAAV profiles between the two cell lines (Fig. 4D). To confirm the conclusion about SAAV proteoform profile differences, we further analyzed the SAAV containing proteoforms from 1D CZE-MS/MS (fig. S6). Although the number of SAAV proteoforms from SW620 cells is about twice as many as that from SW480 cells, only half of the SW480 SAAV proteoforms are covered by the SW620 ones. Manual evaluation of some SAAV proteoforms exclusively identified from SW480 and SW620 cells in raw MS data supported the conclusion. Figure S7 shows the EIEs of one Tumor suppressor p53 (TP53) proteoform containing SAAV from triplicate measurements of SW480 and SW620 cells. The TP53 proteoform was only identified in SW620 cells via MS/MS, and its base peak intensity in SW620 cells was about eightfold higher than that in SW480 cells (5.6 ± 0.6 × 10^4^ versus 0.7 ± 0.3 × 10^4^).

Figure 4E shows the sequences and fragmentation patterns of two examples of proteoforms containing SAAVs. TP53 is an important tumor suppressor closely related to CRC development, and it is an essential member in WNT/β-catenin signaling and PI3K/AKT signaling pathways. We identified one TP53 proteoform containing an AAV at position 72 (P → R) due to the codon 72 polymorphism. Studies have shown the functional differences of the P72 and R72 proteoforms of TP53 (*46*, *47*). For example, the R72 proteoform does a markedly better job of inducing apoptosis compared to the P72 proteoform (*46*). Another study indicated that the expression of P72 proteoform increased CRC metastasis and that the R72 proteoform does not exist in the nonmetastatic CRC cell line (SW480) based on the nucleic acid data (*47*). We only identified the R72 proteoform of TP53 in the SW620 cell line, not in the SW480 cell line, from the top-down MS data. *MSH6* is one of the DNA mismatch repair genes, and its mutations play a crucial role in Lynch syndrome, which is an inherited form of CRC. We identified one DNA mismatch repair protein Msh6 (MSH6) proteoform containing an SAAV due to polymorphism at position 39 (G → E). The G39E SAAV has been associated with an increased risk of CRC according to the nucleic acid data (*48*). We identified G39 proteoforms of MSH6 in both SW480 and SW620 cells but identified the E39 proteoform only in the SW480 cells not in the SW620 cells.

For the proteoforms containing SAAVs, we further performed QIAGEN IPA of the corresponding 82 genes. We revealed that 75 of those genes are associated with tumorigenesis of tissue (*P* value: 0.0001), and three genes (*MSH6*, *PITX1*, and *TP53*) relate to the development of colon tumor (*P* value: 0.002). Five of the genes related to tumorigenesis of tissue (*AURKA*, *EIF5A*, *PFKFB3*, *POLE4*, and *TP53*) are targets of cancer drugs. We further performed IPA network analysis and revealed that 17 of the 82 genes are involved in a cancer-related network (network score, 36) (Fig. 4F), suggesting their crucial roles in cancer and development. The 17 genes are highlighted in purple, and those proteins belong to several different families, including enzyme (diamond shape: *LARS1*, *PARS1*, *ALDOA*, *MSH6*, and *PPIF*), phosphatase/kinase (triangle shape: *PGAM1*, *SET*, and *PFKFB3*), transcription regulator (oval shape: *TP53* and *PITX1*), and others (circle shape: *PSG1*, *SRP14*, *MAGEB2*, *MT1G*, *MT1H*, *MT1M*, and *ISG15*). Nine of those highlighted proteins have direct (solid line) or indirect (dotted line) interactions with TP53.

### Quantitative TDP of metastatic and nonmetastatic human CRC cell lines

**Fig. 5.**
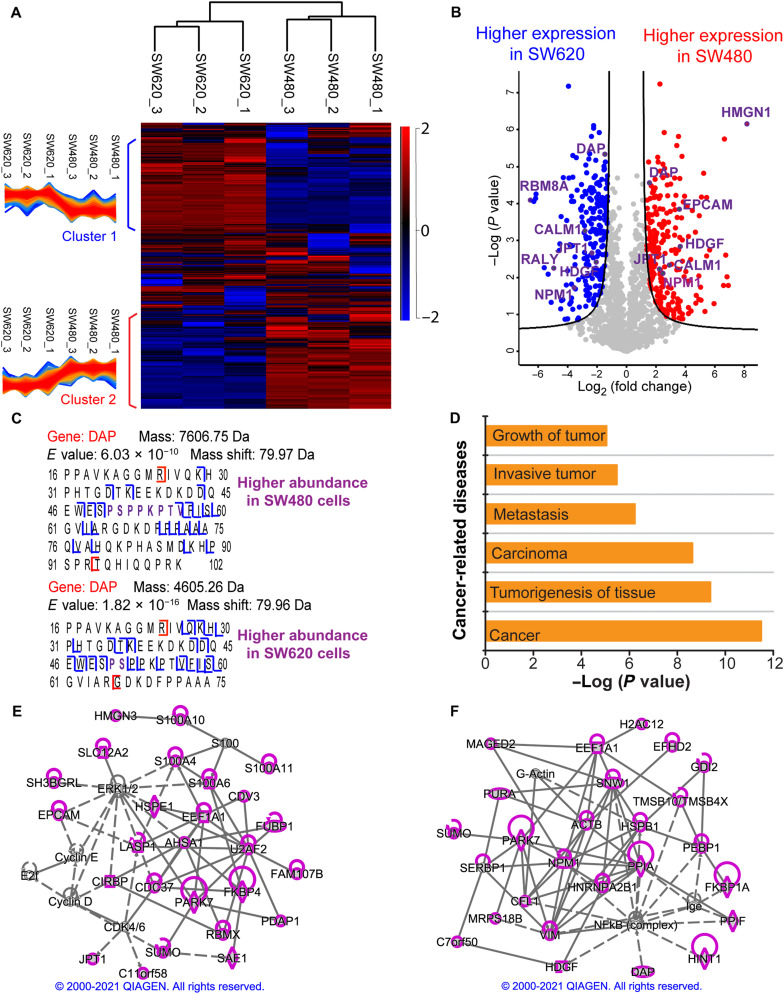
Summary of the LFQ data of SW480 and SW620 cells from SEC-CZE-MS/MS in technical triplicate. (**A**) Heatmap and cluster analysis of the quantified proteoforms (~1500 proteoforms) regarding LFQ intensities. A *z*-score normalization was used. The red color represents high intensity, and the blue color indicates low intensity. (**B**) Volcano plot showing differentially expressed proteoforms between the two cell lines. The quantified proteoforms (~1500) were used for the analysis. Red dots and blue dots represent proteoforms having statistically significantly higher abundance in SW480 and in SW620, respectively. Gene names of some differentially expressed proteoforms are labeled. The Perseus software was used for generating the heatmap in (A) and volcano plot in (B) with the following settings (S0 = 1 and FDR = 0.05) (*49*). (**C**) Sequences and fragmentation patterns of two phosphorylated proteoforms of the gene *DAP*. One has higher abundance in SW480 cells, and the other has higher expression in SW620 cells. (**D**) An IPA analysis reported some cancer-related diseases that are related to the differentially expressed genes in the two cell lines. Proteoforms with higher abundance in SW480 cells (**E**) or higher abundance in SW620 cells (**F**) correspond to genes that are involved in cancer-related networks with high scores. Those genes are highlighted in purple. The diamond, oval, hexagon, trapezium, square, and circle shapes represent enzyme, transcription regulator, translation regulator, transporter, growth factor, and others. The solid and dotted lines represent direct and indirect interactions. Copyright permission has been granted by QIAGEN for using the network data.

We further carried out the first quantitative TDP study of a pair of metastatic (SW620) and nonmetastatic (SW480) human CRC cell lines. The cell lysates of SW480 and SW620 cells were fractionated by SEC, and each fraction was analyzed by CZE-MS/MS in technical triplicate. After database search with TopPIC, we identified roughly 4000 proteoforms per replicate per cell line with a 1% proteoform-level FDR. The overall intensity distributions of identified proteoforms across technical triplicates and the two cell lines are consistent (fig. S8). We performed LFQ analysis using TopDiff (version 1.3.4), a tool in the TopPIC suite, which reported about 1500 proteoforms with measured intensities in all the six samples (three replicates per cell line and two cell lines). The SEC-CZE-MS/MS system shows a reasonably good reproducibility regarding the intensities of shared proteoforms, as evidenced by the strong linear correlations of proteoform intensities between technical replicates of SW480 or SW620 cells (Pearson correlation coefficients: 0.86 to 0.93; fig. S9). The Pearson correlation coefficients of proteoform intensity between SW480 and SW620 cells are statistically significantly lower than that between technical replicates of one cell line (0.71 ± 0.01 versus 0.90 ± 0.03, *P* < 10^−10^, two-tailed Student’s *t* test), indicating substantial differences between the two cell lines in terms of proteoform intensity. We used the Perseus software for further data analysis (*49*). The two cell lines can be easily distinguished using the proteoform quantification profiles (Fig. 5A). Two clusters of differentially expressed proteoforms across the six samples were revealed.

According to the volcano plot in Fig. 5B, 460 proteoforms of 248 proteins showed statistically significant differences in abundance between the two cell lines (FDR < 0.05). Specifically, 244 proteoforms of 152 proteins had higher abundance in the SW480 cell line, and 216 proteoforms of 132 proteins had higher expression in the SW620 cell line. Figure 5B shows that one HMGN1 proteoform and one RBM8A proteoform have the most significant abundance changes between SW480 and SW620 cells. HMGN1 regulates gene expression and PTMs of core histones, affecting DNA repair and tumor progression (*50*). It has been reported that RBM8A promotes tumor cell migration and invasion in the most common type of primary liver cancer (*51*).

Comparing the overexpressed and underexpressed proteoforms in the two cell lines revealed that 36 genes (e.g., *DAP*, *CALM1*, *HDGF*, *JPT1*, and *NPM1*) have both overexpressed and underexpressed proteoforms in one cell line, suggesting that different proteoforms of the same gene had completely different expression patterns in the two cell lines. Figure 5C shows two differentially expressed proteoforms of *DAP* (death-associated protein 1), one of those 36 genes. It has been reported that DAP modulates cell death and correlates with the clinical outcome of patients with CRC (*52*). We revealed that one phosphorylated proteoform of DAP (~7607 Da, phosphorylation site S51 or T56) had a higher abundance in SW480 cells, and another phosphorylated proteoform (~4605 Da, phosphorylation site S51) showed higher expression in SW620 cells. Both the S51 and T56 are known to be phosphorylated according to PhosphoSitePlus, with S51 being the most common phosphorylation site of DAP. We noted that the differentially expressed proteoforms in this study include phosphorylated proteoforms of several important genes related to CRC, i.e., *RALY* (*53*), *NPM1* (*54*), *DAP* (*52*), and *HDGF* (*55*) (table S2). The functions of phosphorylated forms of those four proteins in modulating CRC development are still unclear. However, the differential expressions of those phosphorylated proteoforms in the metastatic and nonmetastatic CRC cells suggest their potential roles in regulating CRC metastasis. We also manually checked the MS raw data of three of the phosphorylated proteoforms in table S2 (*NPM1*, *RALY*, and *HNRNPC*), and their EIEs are shown in figs. S10 to S12. The results clearly indicate their significantly higher abundance in SW620 cells compared to SW480 cells, agreeing well with the data in table S2.

We highlight several differentially expressed proteoforms of CALM1, Jupiter microtubule associated homolog 1 [JPT1 (HN1)], and Epithelial cell adhesion molecule (EPCAM) . CALM-dependent systems play important roles in cancer metastasis (*56*). JPT1 (HN1) promotes cancer metastasis via activating the nuclear factor κB (NF-κB) signaling pathway (*57*). EPCAM is a human cell surface glycoprotein and plays crucial roles in tumor biology, especially CRC (*58*). EPCAM has been recognized as an important therapeutic target for cancer. We found two CALM1 proteoforms having significantly higher abundance in SW620 cells compared to SW480 cells; one of them contains K116 trimethylation. We revealed one CALM1 proteoform showing higher abundance in SW480 cells, and the proteoform carries N-terminal acetylation and a 58-Da mass shift between amino acid residues 73 and 89. The 58-Da mass shift can be explained as a trimethylation/acetylation plus oxidation. Three of JPT1 proteoforms have higher abundance in SW480 cells, and one of them contains a 167-Da mass shift between the amino acid residues 66 and 89, where seven serine residues can be phosphorylated according to the PhosphoSitePlus database (www.phosphosite.org/). The 167-Da mass shift most likely represents a combination of phosphorylation and other PTMs. One JPT1 proteoform shows higher abundance in SW620 cells. We also observed two EPCAM proteoforms having higher abundance in SW480 cells.

We then performed IPA analyses of the genes of those differentially expressed proteoforms between SW480 and SW620 cells. Those genes are heavily involved in cancer-related diseases, for example, tumorigenesis of tissue and metastasis (Fig. 5D). Five of those proteins [Eukaryotic Translation Initiation Factor 4E (EIF4E), EPCAM, Peptidyl-prolyl cis-trans isomerase (FKBP1A), Lysosomal alpha-glucosidase (GAA), and Heat shock protein HSP 90-beta (HSP90AB1)] are drug targets. IPA network analyses revealed that 26 proteins (highlighted in purple) whose proteoforms showed higher abundance in SW480 compared to SW620 were involved in a cancer-related network (score, 51) (Fig. 5E). Those proteins belong to several families, including enzyme (diamond shape, e.g., PARK7 and FKBP4), transcription regulator (oval shape, e.g., FUBP1), translation regulator (hexagon shape, e.g., CIRBP and EEF1A1), transporter (trapezium shape, e.g., SLC12A2 and LASP1), and others (circle shape, e.g., EPCAM and JPT1). Most of those proteins have direct (solid line) and indirect (dotted line) interactions with one another. We also carried out network analysis for the proteins whose proteoforms had higher expression in SW620 cells and observed high scores for cancer-related networks. Figure 5F shows one cancer-related network (score, 54), and 26 of those proteins are involved in the network (highlighted in purple). Those proteins include several CRC-related important proteins, Nucleophosmin (NPM1) (oval shape, transcription regulator, located in the nucleus), DAP (transcription regulator, located in the cytoplasm), and Hepatoma-derived growth factor (HDGF) (square shape, growth factor, located in the extracellular space). NPM1 is a crucial protein in the network, and many of the highlighted proteins have direct interactions (solid line) with NPM1, for example, Parkinson disease protein 7 (PARK7), Vimentin (VIM), and Peptidyl-prolyl cis-trans isomerase A (PPIA). NPM1 also has indirect interaction (dotted line) with the NF-κB complex, which plays crucial roles in modulating DNA transcription and cell survival. Human NPM1 boosts the activation of NF-κB according to ingenuity relationships from the IPA analysis. Besides NPM1, several other highlighted proteins (e.g., HDGF and DAP) also have indirect interactions with the NF-κB complex. For example, NF-κB regulates the transcription of *HDGF*, and DAP deactivates the NF-κB according to the IPA network analysis results. The IPA analysis also revealed that 13 proteoforms of three genes (*EIF4B*, *EIF4E*, and *EIF4EBP1*) in the mTOR signaling pathway had statistically significant differences in abundance between the SW480 and SW620 cells (data file S1).

## DISCUSSION

TDP is facing technical challenges for deep proteoform profiling of human cells. Although remarkable technical progresses have been achieved in LC-MS/MS–based TDP during the past two decades, the number of proteoform IDs per human cell line has been stabilized on the level of 3000 for a decade (*18*–*22*). Alternative strategies for deep TDP of human cells are needed. CZE-MS/MS has been recognized as one alternative strategy for TDP (*23*, *29*, *30*, *59*), most likely due to the high separation efficiency of CZE and high sensitivity of CZE-MS for proteoform separation and detection. However, the performance of CZE-MS/MS for TDP profiling of human cell proteoforms is limited due to the extremely high sample complexity and limited sample loading capacity of CZE, which is evidenced by the 1D CZE-MS/MS data of CRC cells in this work and our previous work with the ID of only hundreds of human proteoforms in one run (*24*). In this study, we advanced TDP of human cells drastically in terms of the number of proteoform IDs per human cell line compared to previous LC-MS/MS–based studies (~16,000 versus ~3000) via coupling LC fractionations to CZE-MS/MS. This work represents an important progress in TDP, which aims to characterize the human proteome in a proteoform-specific manner (Human Proteoform Project) (*16*). We need to highlight that SEC-CZE-MS/MS and RPLC-CZE-MS/MS under optimized conditions will be powerful analytical techniques for deep TDP of human cells with high throughput (Fig. 2A). CZE-MS/MS analyses of only six SEC fractions of a SW480 cell lysate produced about 4000 proteoform IDs and roughly 700 proteoform IDs per CZE-MS/MS run. The data indicate that it is feasible now using LC-CZE-MS/MS (i.e., SEC-CZE-MS/MS) for deep TDP profiling of a large number of human cell types, which will potentially transform basic and translational biomedical research. The MS raw data have been deposited to the ProteomeXchange Consortium via the PRIDE (*60*) partner repository with the dataset identifier PXD029703.

TDP of metastatic and nonmetastatic cells is crucial for discovering new protein biomarkers and providing a more accurate understanding of molecular mechanisms of cancer metastasis. According to the results from our qualitative and quantitative TDP of SW480 and SW620 cells, we had several conclusions about CRC metastasis. First, CRC cells have a drastic transformation in proteoforms and SAAVs after metastasis, evidenced by obvious differences of proteoform and SAAV profiles between SW480 and SW620 cells. Second, different proteoforms from the same cancer-related gene (e.g., *DAP*, *CALM1*, *HDGF*, *JPT1*, *RALY*, and *NPM1*) may have potentially varied biological functions in modulating CRC metastasis, because they show opposite expression profiles between the SW480 and SW620 cells (Fig. 5B). Some proteoforms of those genes have higher abundance in SW480 cells; some of their proteoforms show higher expression in SW620 cells. Third, PTMs (i.e., phosphorylation) of important cancer-related genes (i.e., *DAP*, *HDGF*, *JPT1*, *RALY*, *NPM1*, *MARK2*, *SOX9*, *EIF4B*, and *EIF4EBP1*) could play important roles in regulating CRC metastasis, evidenced by the significant abundance differences of phosphorylated proteoforms from those genes between the SW480 and SW620 cells. The differentially expressed proteoforms, especially those with PTMs, of important cancer-related genes could be novel proteoform biomarkers of CRC metastasis. Fourth, proteoforms of genes in well-known CRC-related pathways (WNT/β-catenin signaling, PI3K/AKT signaling, mTOR signaling, and ERK/MAPK signaling) are different between SW480 and SW620 cells, and those proteoforms could play vital roles in modulating CRC metastasis.

Our TDP strategies still have some technical limitations. One relates to the ID of large proteoforms. In this work, we focused on the characterization of proteoforms smaller than 30 kDa. CZE-MS/MS has a much lower sample loading capacity compared to RPLC-MS/MS (nanoliters versus microliters), resulting in a limited mass of protein materials that can be injected for measurements with CZE-MS/MS. This issue is particularly severe for the characterization of large proteoforms in a complex proteome sample because large proteoforms tend to have drastically lower signal-to-noise ratios than small proteoforms due to the much wider charge state distributions. Highly efficient size-based fractionation techniques must be used to enrich large proteoforms before CZE-MS/MS. In addition, more effort needs to be made to improve the sample loading capacity of CZE-MS/MS via investigating online sample stacking techniques or solid phase microextraction methods. Another limitation relates to the extensive fragmentation of proteoforms for accurate localization of PTMs. The backbone cleavage coverage of proteoforms from commonly used collision-based fragmentation techniques [i.e., collision-induced dissociation and higher energy collision dissociation (HCD)] is limited. We expect that coupling our LC-CZE-MS/MS technique to a mass spectrometer with electron- or photon-based gas-phase fragmentation techniques [i.e., electron-capture dissociation (*61*), electron-transfer dissociation (*62*), and ultraviolet photodissociation (*63*)] will revolutionize TDP for the Human Proteoform Project (*16*).

## MATERIALS AND METHODS

### Materials and reagents

MS-grade water, acetonitrile (ACN), methanol, formic acid (FA), and high-performance liquid chromatography (HPLC)–grade acetic acid were purchased from Thermo Fisher Scientific (Pittsburgh, PA). Ammonium bicarbonate (NH_4_HCO_3_), urea, dithiothreitol (DTT), iodoacetamide (IAA), and 3-(trimethoxysilyl)propyl methacrylate were from Sigma-Aldrich (St. Louis, MO). Hydrofluoric acid (HF; 48 to 51% solution in water) and acrylamide were purchased from Acros Organics (NJ, USA). Fused silica capillaries (inner diameter, 50 μm/outer diameter, 360 μm) were purchased from Polymicro Technologies (Phoenix, AZ). cOmplete, Mini protease inhibitor cocktail (EASYpacks) was from Roche (Indianapolis, IN).

### Sample preparation

SW480 (catalog no. CCL-228) and SW620 (catalog no. CCL-227) original cell lines were both purchased from American Type Culture Collection (Manassas, VA) and were cultured in RPMI 1640 cell culture medium (Life Technologies Corporation, Grand Island, NY) supplemented with 10% fetal bovine serum (Thermo Fisher Scientific, Gaithersburg, MD) and 2 mM l-glutamine (Invitrogen, San Diego, CA). The cells were incubated at 37°C with 5% CO_2_ and were passaged every 3 to 4 days. Both cell lines were last verified by short tandem repeat sequencing in 2016 and were used within 2 months after resuscitation from frozen aliquots at −80°C.

Upon growing to confluency, cells were harvested and cleansed of the remaining cell culture medium via subsequent washing with HPLC-grade water (Thermo Fisher Scientific, Pittsburgh, PA) and centrifugation for 5-min intervals at 15,000*g* until the supernatant was clear. Proteins were then extracted using mammalian cell lysis buffer. Cell lysis buffer consisted of 8 M urea, 50 mM tris (pH 8.2), 1 mM β-glycerophosphate, 1 mM phenylmethylsulfonyl fluoride, 75 mM sodium chloride, 1 mM sodium fluoride, 1 mM sodium orthovanadate, 10 mM sodium pyrophosphate, and one protease inhibitor cocktail. The reagents for cell lysis buffer were purchased from Sigma-Aldrich, and cOmplete EDTA-free protease inhibitor cocktail tablet was purchased from Roche. The lysis buffer was added to the harvested cells, which then underwent sonication on ice three times for 1-min intervals at 15% amplitude. The resulting extracted proteins were then clarified of cellular debris by centrifugation at 15,000 rpm for 10 min. Proteins were quantified using a bicinchoninic acid protein assay (Thermo Fisher Scientific Pierce, Rockford, IL) and then stored at −80°C until preparation for MS analysis.

SW480 and SW620 proteins were denatured at 37°C for 30 min, reduced at 37°C for 30 min using DTT, and then alkylated at room temperature in the dark for 20 min using IAA. The excess IAA were quenched by adding DTT and reacting for 5 min at room temperature.

For experiment 1 (RPLC-CZE-MS/MS), 200 μg of proteins from SW480 and SW620 cells was reduced, alkylated, and acidified, followed by RPLC fractionation into 13 fractions and CZE-MS/MS. For experiment 2 (SEC-RPLC-CZE-MS/MS), 2 mg of proteins from SW480 and SW620 cells was reduced and alkylated before fractionated by SEC-RPLC and analyzed by CZE-MS/MS. For experiment 3 (RPLC-CZE-MS/MS), 420 μg of proteins from SW480 and SW620 cells was reduced and alkylated before fractionation by RPLC into six fractions and analyzed by CZE-MS/MS. For experiment 4 (SEC-CZE-MS/MS), the samples were desalted after reduction and alkylation using a C4 trap column (4 mm by 10 mm, 3-μm particles, 300-Å pore size). Specifically, 500 μg of proteins from SW480 and SW620 cells was loaded onto the column and flushed with mobile phase A (MPA) [2% (v/v) ACN and 0.1% FA] for 10 min at a flow rate of 1 ml/min. The proteins were eluted with MPB (80% ACN and 0.1% FA) for 3 min at flow rate of 1 ml/min. The eluates were lyophilized with a speed vacuum and redissolved in 150 μl of 0.1% FA. Then, proteins from SW480 and SW620 cells were fractionated by SEC into six fractions, followed by CZE-MS/MS analyses. For experiment 5 (1D CZE-MS/MS), 100 μg of proteins from SW480 and SW620 cells was desalted using two methods. In one case, both samples were desalted by a C4 trap column as described in experiment 4. In the other case, both samples were desalted by Amicon Ultra centrifugal filters with a molecular weight cutoff of 10 kDa. Desalting with centrifugal filter was performed by loading 100 μg of proteins onto the filter and washing the sample four times with 50 mM NH_4_Ac at 14,000*g*. Last, the sample was recovered in 30 μl of 50 mM NH_4_Ac. The samples desalted with the C4 trap column, and centrifugal filters were analyzed by 1D CZE-MS/MS in technical triplicate.

### Fractionation of the SW480 and SW620 proteome

All separations were performed on a 1260 Infinity II HPLC system from Agilent (Santa Clara, CA). Detection was performed using an ultraviolet-visible detector at a wavelength of 254 nm. Data were collected and analyzed using OpenLAB software. RPLC (C4, 2.1 mm by 250 mm; Sepax Technologies) and SEC (4.6 mm by 300 mm, 500-Å pores; Agilent) were performed offline (Agilent HPLC) for prefractionation. Fractions from SW620 and SW480 from experiment 1 (13 fractions × 2 samples), experiment 2 (84 fractions × 2 samples), experiment 3 (6 fractions × 2 samples), and experiment 4 (6 fractions × 2 samples) were analyzed by CZE-MS/MS, respectively.

In experiment 1, RPLC was used for sample fractionation with a flow rate of 0.25 ml/min and a gradient of 0 to 80% MPB over 90 min (MPA: 2% ACN and 0.1% FA in water; MPB: 80% ACN and 0.1% FA in water). Fractions were collected from 15 to 22 min (fraction 1) and 22 to 70 min (12 fractions, 4 min per fraction). For experiment 2, both SEC and RPLC were used for fractionation before CZE-MS/MS. For SEC, the flow rate was 0.35 ml/min with a 0.05% trifluoroacetic acid (TFA) MP. Two milligrams of proteins in an 800-μl solution was fractionated by SEC. Fractions were collected from 5 to 8 min (fraction 1) and 8 to 12.5 min (3 fractions, 1.5 min per fraction). One RPLC run was performed for each SEC fraction with a flow rate of 0.25 ml/min and a gradient of 0 to 80% MPB (MPA: 2% ACN and 0.1% TFA in water; MPB: 10% IPA and 0.1% TFA in ACN) over 90 min with a 10-min equilibration with 100% MPA at the beginning of the separation. Fractions were collected from 20 to 25 min (fraction 1) and 25 to 65 min (20 fractions, 2 min per fraction). In experiment 3, RPLC fractionation was carried out using the same MPs as in experiment 1, and a 90-min gradient was used with a 10-min equilibration with 100% MPA at the beginning of the separation. Fractions were collected from 25 to 55 min (fraction 1), 50 to 70 min (4 fractions, 5 min per fraction), and 70 to 95 min (fraction 6). In experiment 4, SEC fractionation was performed with an Agilent Bio SEC-5 column (4.6 mm by 300 mm, 5-μm particles, 500-Å pore size). Two hundred twenty micrograms of SW480 and SW620 proteins (1.5 mg/ml, 75 μl × 2 injections) was loaded into the SEC column and separated isocratically at a flow rate of 0.3 ml/min with 0.1% FA as MP. The first fraction is collected from 5.6 to 8.6 min. The second to the fifth fraction was from 8.6 to 14.6 min with 1.5 min per fraction. The final fraction was collected from 14.6 to 19.0 min. In experiments 1 to 4, samples were dried down and redissolved in 50 mM NH_4_HCO_3_ (pH 8.0, ~2 mg/ml) for CZE-ESI-MS/MS.

### CZE-MS/MS analysis

CZE separation was performed using a CESI 8000 Plus CE system (Beckman Coulter). A commercialized electrokinetically pumped sheath-flow Capillary Electrophoresis (CE)-MS nanospray interface (CMP Scientific Corp.) was applied for online coupling the CE system and mass spectrometer (*64*, *65*). A glass emitter (orifice size: 20 to 30 μm) installed on the interface was filled with sheath buffer (0.2% FA and 10% methanol) to generate electrospray at voltage of 2 to 2.3 kV.

A 100-cm lysophosphatidic acid (LPA)–coated fused silica capillary (inner diameter, 50 μm; outer diameter, 360 μm) was used for CZE separation in experiments 1, 2, 4, and 5, while a 70-cm LPA-coated capillary (inner diameter, 50 μm; outer diameter, 360 μm) was used for separation in experiment 3. The inner wall of the capillary was coated with LPA based on the procedure described in (*66*). One end of the capillary was etched with HF to reduce the outer diameter of the capillary to about 70 to 80 μm based on the procedure described in (*67*). (Caution: Use appropriate safety procedures while handling HF solutions).

In experiments 1, 2, 4, and 5, the capillary (100 cm) was loaded with 500 nl of sample. In experiment 3, the capillary (70 cm) was loaded with ~350 nl of sample. After sample loading, the capillaries were inserted into background electrolyte, containing 5% acetic acid (pH 2.4), and a 30-kV voltage was applied at the sample injection end to carry out separations.

MS1 and MS2 data were collected on a Q-Exactive HF mass spectrometer (Thermo Fisher Scientific) under a data-dependent acquisition mode. The temperature of ion transfer tube was set to 320°C, and s-lens radio frequency (RF) was 55. MS1 spectra were collected with the following parameters: an *m*/*z* range of 600 to 2000, a mass resolution of 120,000 (at *m*/*z* of 200), a microscan number of 3, an Automatic gain control (AGC) target value of 1 × 10^6^, and a maximum injection time of 100 ms. The top five most abundant precursor ions (charge state higher than 5 or charge state unassigned and intensity threshold 2 × 10^4^) in the MS1 spectra were isolated with a window of 4 *m*/z and fragmented via HCD with Normalized collision energy (NCE) of 20%. The settings for MS2 spectra were a resolution of 120,000 (at *m*/*z* of 200), a microscan number of 3, an AGC target value of 1 × 10^5^, and a maximum injection time of 200 ms. The dynamic exclusion was set to a duration of 30s, and the isotopic peaks were excluded.

In experiments 2 to 5, each LC fraction was analyzed by CZE-MS/MS in triplicate. In experiment 1, each LC fraction was analyzed by a single CZE-MS/MS run. In total, 410 MS raw files with good protein signals were produced from experiments 1 to 4 for database search, including 26 MS raw files from experiment 1 (13 fractions × 2 samples), 312 MS raw files from experiment 2 (52 fractions × 2 samples × 3 replicates), 36 MS raw files from experiment 3 (6 fractions × 2 samples × 3 replicates), and 36 MS raw files from experiment 4 (6 fractions × 2 samples × 3 replicates). We need to note that we collected 84 fractions × 2 samples in experiment 2. However, we only observed good protein signals from 52 LC fractions per sample. Twelve MS RAW files were collected from experiment 5 using CZE-MS/MS.

### Data analysis for proteoform ID

All RAW files were analyzed with the TopPIC Suite (version 1.4.0) pipeline (*15*, *68*). The RAW files were converted into mzML files with msconvert (*69*). Then, spectral deconvolution was performed with TopFD (version 1.4.0), which converts precursor and fragment isotope clusters into neutral monoisotopic masses and finds proteoform features by combining precursor isotope clusters with similar monoisotopic masses and close migration times in MS1 scans. The resulting mass spectra with monoisotopic neutral masses were stored in msalign files, and the proteoform feature information was stored in text files. The human proteome database was downloaded from UniProt (UP000005640, 20,350 entries, version 23 October 2019, only reviewed protein sequences were included) and concatenated with a random decoy database of the same size. Each msalign file was searched against the concatenated targe-decoy database using TopPIC (version 1.4.0). Cysteine carbamidomethylation was set as a fixed modification, and the maximum number of unexpected modifications was 1. The precursor and fragment mass error tolerances were 15 parts per million. The maximum mass shift of unknown modifications was 500 Da. TopPIC reported a list of target and decoy PrSMs for each msalign file.

The proteoforms identified from all msalign files were merged and filtered with a proteoform-level FDR. First, the target and decoy PrSMs reported from all the msalign files were combined and filtered with a 5% spectrum-level FDR. The PrSMs were then clustered by grouping PrSMs into the same cluster if they were from the same protein and their precursor mass differences were not large than 2.2 Da. The PrSM with the best E-value was selected for each cluster, and its proteoform was reported as the representative one for the cluster. The representative target and decoy proteoforms were lastly filtered with a 1% proteoform-level FDR.

### Proteoform quantification

There were 18 MS raw files from triplicate CZE-MS/MS analyses of the six SEC fractions for the SW480 or SW620 sample in experiment 4. The TopPIC suite pipeline reported a list of target and decoy PrSM IDs for each raw file. Using the methods in the previous section, the PrSM IDs of the 36 MS raw files were merged, and a list of proteoform IDs with a 1% proteoform-level FDR was reported. The abundance of a proteoform was computed as the sum of the proteoform abundances in the six SEC fractions, which were reported by TopFD. Proteoform IDs and their abundances were reported for each replicate using this method. Last, TopDiff (version 1.4.0), a tool in TopPIC Suite, was used to match proteoform IDs across the three SW480 replicates and three SW620 replicates.

The quantitative results were further analyzed using Perseus software (*49*). The intensities of each proteoform in triplicate CZE-MS/MS runs of SW480 and SW620 were normalized to the intensity of corresponding proteoform from the first run of SW480, converting proteoform intensity to proteoform ratio. Then, proteoform ratios of each run were divided by the corresponding median to make sure the ratios center at 1. After log_2_ transformation of all the data, the significantly differentially expressed proteoforms were determined by performing *t* test analysis (FDR threshold: 0.05; S0: 1) using the Perseus software. The volcano plot [−log (*P* value) versus log_2_ (fold change)] was generated.

### Proteogenomic analysis

To generate sample-specific protein sequence databases with genetic variations for SW480 and SW620 cells, two RNA-seq datasets (SRR8616059 for SW480 and SRR8615459 for SW620) (*70*) were downloaded from the Sequence Read Archive. The Genome Analysis Toolkit (GATK) pipeline (*71*) was used to align short reads in the RNA-seq data with the hg38 human genome to call single-nucleotide variants (SNVs) and indels, which were further annotated using the gene-based annotation of ANNOVAR (*72*). The annotated nonsynonymous SNVs and indels in exons were chosen for generating sample-specific protein sequence databases based on the basic annotation of the hg38 human genome in GENCODE (*73*). Two sample-specific protein sequence databases were generated using TopPG (version 1.0) (*45*): one for SW480 cells and the other for SW620 cells. Each protein sequence database contained both reference protein sequences in the basic annotation of GENCODE and protein sequences with sample-specific variants. There were 74,887 entries with 51,485 reference sequences and 23,402 sequences with variants in the database for SW480 cells and 75,665 entries with 51,432 reference sequences and 24,233 sequences with sample-specific variants in the database for SW620 cells. The SW480 and SW620 mass spectra in experiments 3 and 4 were searched against their corresponding sample-specific database using TopPIC (version 1.4.0) with the same parameter setting in the “Data analysis for proteoform ID” section. Using the methods in the “Data analysis for proteoform ID” section, PrSMs identified in each cell line were combined and clustered, and proteoform IDs were filtered by a 5% proteoform-level FDR. IDs with SAAV sites were manually inspected. If a proteoform with SAAV sites contained no unexpected mass shifts or had at least three matched fragment ions between each SAAV site and the unexpected mass shift, then it was reported as a confident proteoform ID with SAAV sites.

### QIAGEN ingenuity pathway analysis

The cancer-related network analysis results shown in Figs. 4F and 5 (E and F) were generated through the use of QIAGEN IPA (QIAGEN Inc.) (*74*). Permissions have been granted by QIAGEN to use those copyrighted figures in this publication.

### Statistical analysis

Data are presented as means ± SDs when available. For the statistical analysis of LFQ data of SW480 and SW620 cell lines, we performed both-sided *t* test using the Perseus software (*49*) to determine the proteoforms with statistically significant abundance difference between the two cell lines with the following settings, S0 = 1 and FDR = 0.05.

## Supplementary Materials

### Other Supplementary Material for this manuscript includes the following:

Lists of identified proteoforms

## Notes

View/request a protocol for this paper from *Bio-protocol*.
