# Supplementary material for: Deep top-down proteomics revealed significant proteoform-level differences between metastatic and nonmetastatic colorectal cancer cells
Source: Sci Adv. 2022 Dec 21;8(51):eabq6348. doi: 10.1126/sciadv.abq6348 (PMC9770947; doi:10.1126/sciadv.abq6348)
Supplement: Supplementary file 1 — Figs. S1 to S12 Tables S1 to S2 [file sciadv.abq6348_sm.pdf]

Science Advances
AAAS

Supplementary Materials for

Deep top-down proteomics revealed significant proteoform-level differences between metastatic and nonmetastatic colorectal cancer cells

Elijah N. McCool *et al.*

Corresponding author: Amanda B. Hummon, hummon.1@osu.edu; Xiaowen Liu, xwliu@tulane.edu; Liangliang Sun, lsun@chemistry.msu.edu

Sci. Adv. **8**, eabq6348 (2022)
DOI: 10.1126/sciadv.abq6348

The PDF file includes:

Figs. S1 to S12
Tables S1 to S2
Legend for lists of identified proteoforms

Other Supplementary Material for this manuscript includes the following:

Lists of identified proteoforms

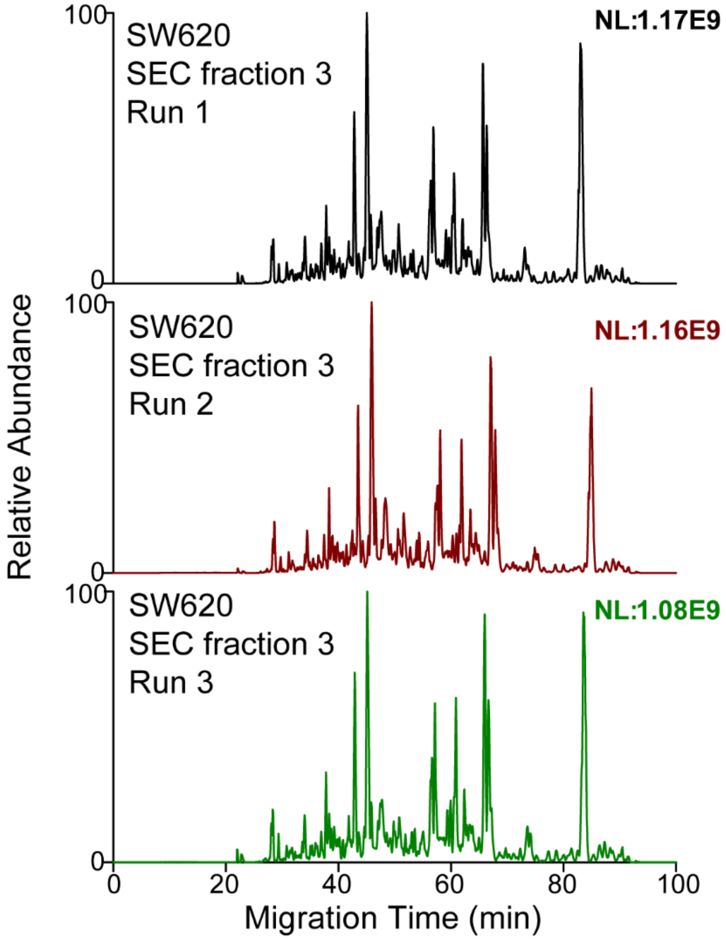

Fig. S1. Base peak electropherograms of one SEC fraction of the SW620 cell lysate after triplicate CZE-MS/MS analyses. The data shows good reproducibility of our CZE-MS/MS system for complex proteome sample analysis.

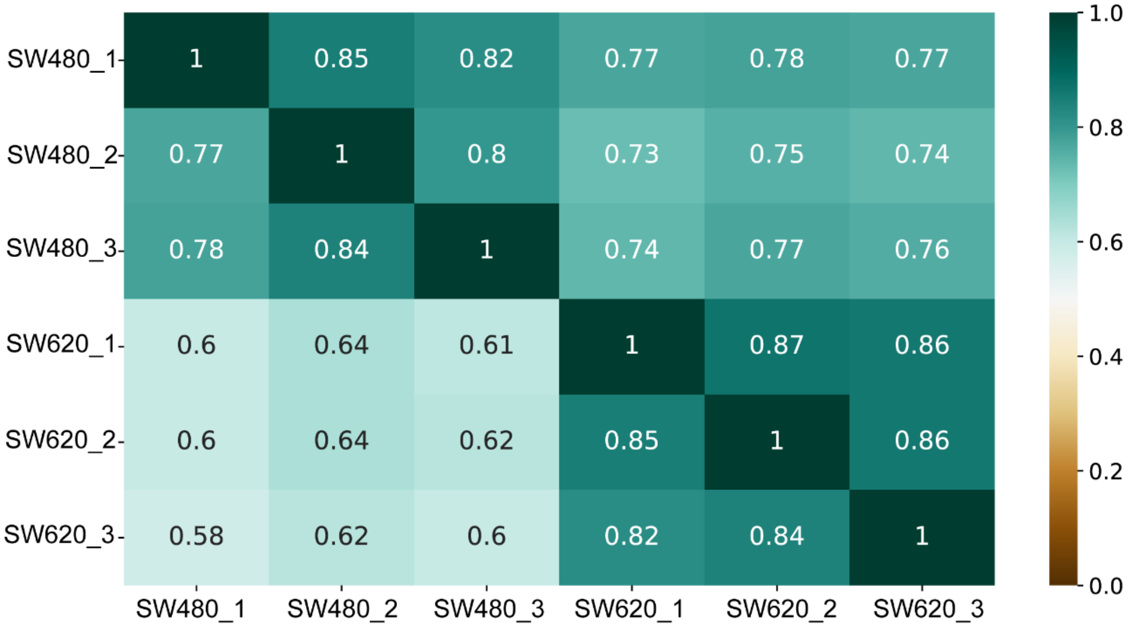

Fig. S2. **Heat map of protein overlaps from technical triplicates of SW480 and SW620 cells using SEC-CZE-MS/MS.** Each number in the figure represents a ratio between the number of shared proteins in two conditions (e.g., SW480_1 (x-axis) and SW620_1 (y-axis)) and the total number of identified proteins in one of the two conditions listed on the y-axis (e.g., SW620_1). For example, the protein overlap between SW480_1 (x-axis) and SW620_1 (y-axis) is 0.6, which indicates the ratio between the number of shared proteins in those two conditions and the total number of identified proteins in SW620_1.

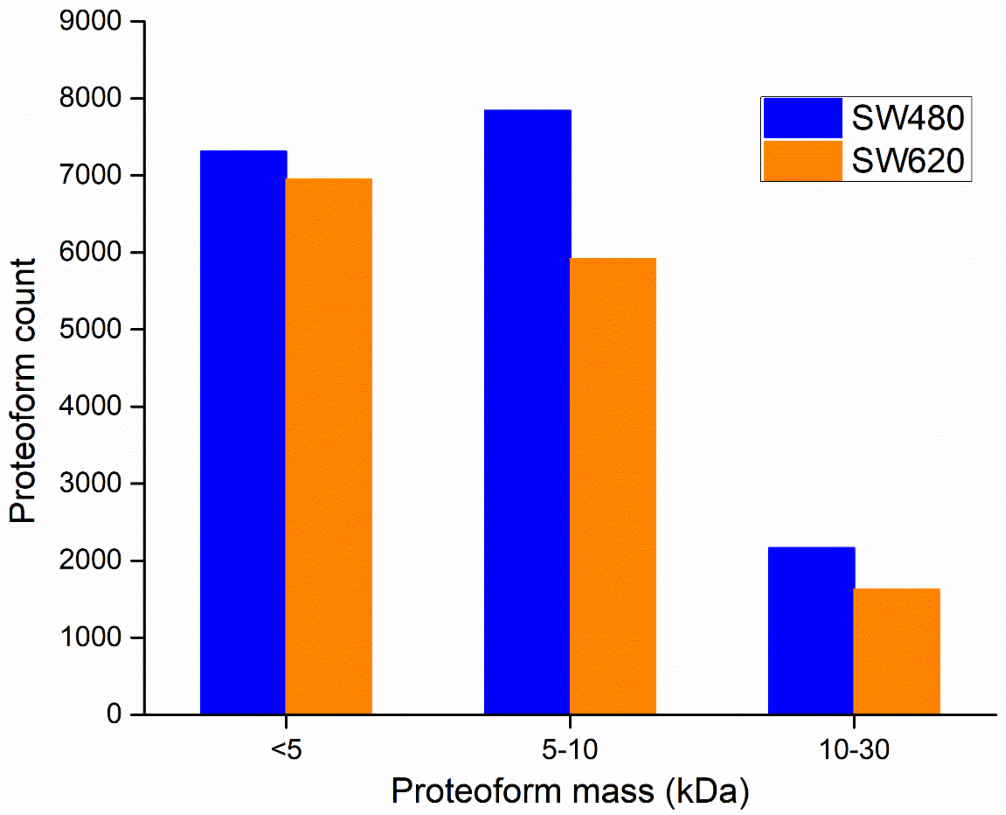

Fig. S3. The mass distribution of identified proteoforms from SW480 and SW620 cells. The data are from the combined results of four CZE-MS/MS-based strategies.

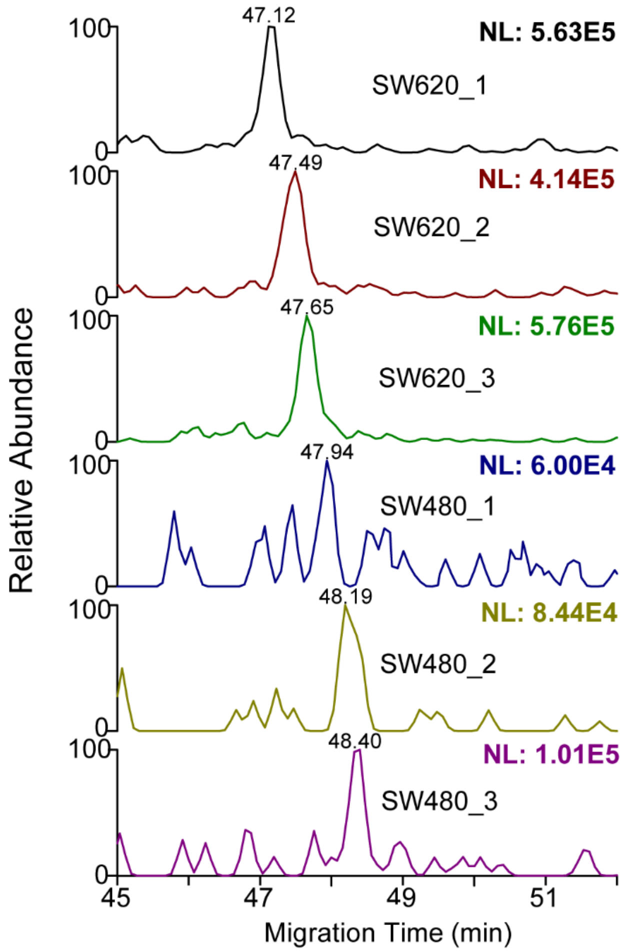

Fig. S4. **Extracted ion electropherograms (EIEs) of one EIF4B phosphorylated proteoform shown in Table 1 from SW480 and SW620 cells after triplicate CZE-MS/MS analyses.** The proteoform sequence is M.AASAKKKNK(KGKTISLTDFL)[mass shift: 122 Da, phosphorylation and acetylation/trimethylation] AEDGGTGGGSTYVSKPVSWADETDDLEGDVSTTWHSNDDDVYRAPPIDRSILPTAPR.A. Precursor m/z corresponding to the identified charge state was extracted using 10 ppm mass tolerance and Gaussian smoothing (5 points). Migration time alignment was performed between the SW480 and SW620 data based on the accurate m/z and charge information.

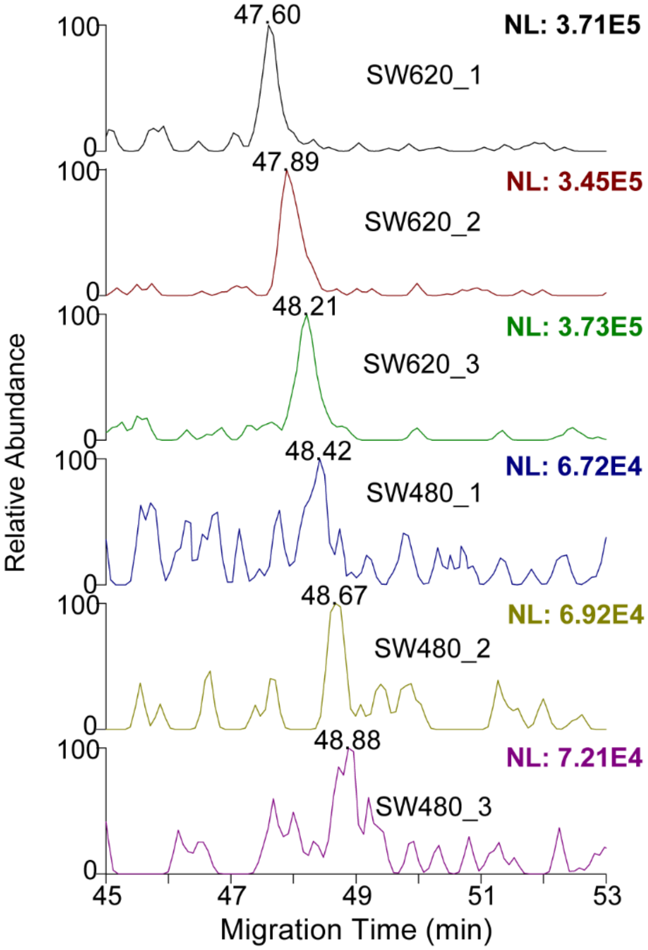

Fig. S5. **Extracted ion electropherograms (EIEs) of one EIF4B phosphorylated proteoform shown in Table 1 from SW480 and SW620 cells after triplicate CZE-MS/MS analyses.** The proteoform sequence is M.(A)[Acetyl]ASAKKKNKKGKTISLTDFLAEDGG(T)[mass shift: 80 Da, phosphorylation]GGGSTYVSKPVSWADETDDLEGDVSTTWHSNDDDVYRAPPIDR.S. Precursor m/z corresponding to the identified charge state was extracted using 10 ppm mass tolerance and Gaussian smoothing (5 points). Migration time alignment was performed between the SW480 and SW620 data based on the accurate m/z and charge information.

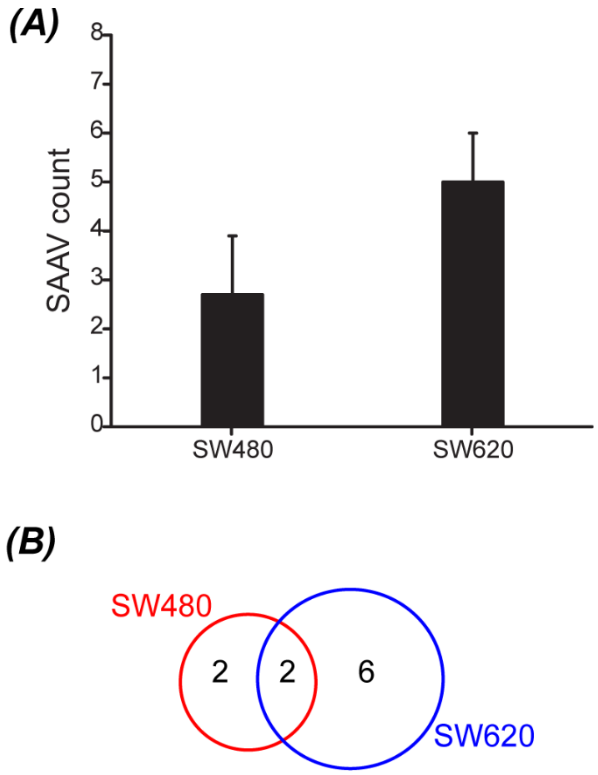

Fig. S6. The SAAV data of SW480 and SW620 cells from 1D CZE-MS/MS. (A) The number of identified proteoforms containing SAAVs. The error bars represent the standard deviations of the number of proteoforms from triplicate measurements. (B) The overlap of SAAV containing proteoforms from 1D-CZE-MS/MS.

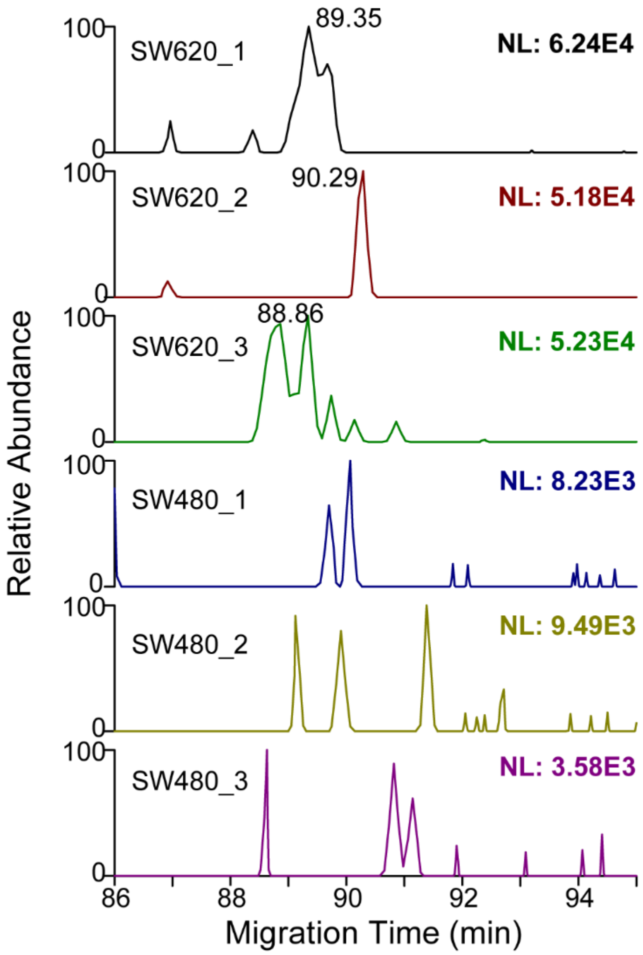

Fig. S7. Extracted ion electropherograms (EIEs) of one TP53 proteoform containing SAAV (K.LLPENNVLSPLPSQAMDDLMLSPDDIEQWFTEDPGPDEAPRMPEAAPR.V) from SW480 and SW620 cells after triplicate CZE-MS/MS analyses. Precursor m/z corresponding to the identified charge state was extracted using 10 ppm mass tolerance and Gaussian smoothing (5 points). Migration time alignment was performed between the SW480 and SW620 data based on the accurate m/z and charge information. The proteoform intensity in SW480 cells is close to the noise and migration times are not labelled.

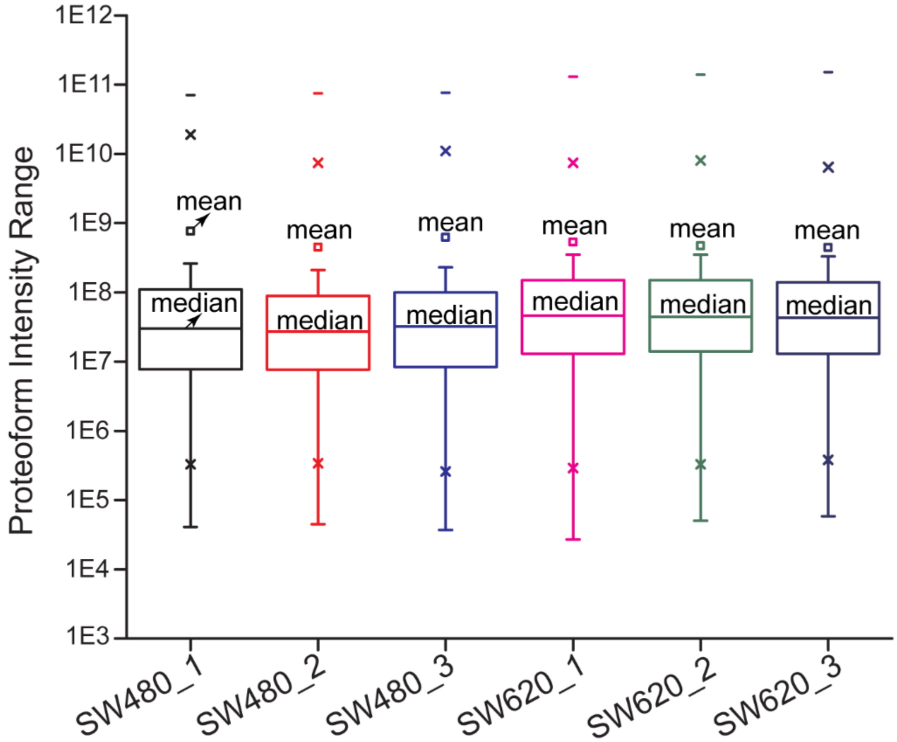

Fig. S8. **Box plots of proteoform intensities from SEC-CZE-MS/MS analyses of SW480 and SW620 cells.** Technical triplicate data of each cell line are shown.

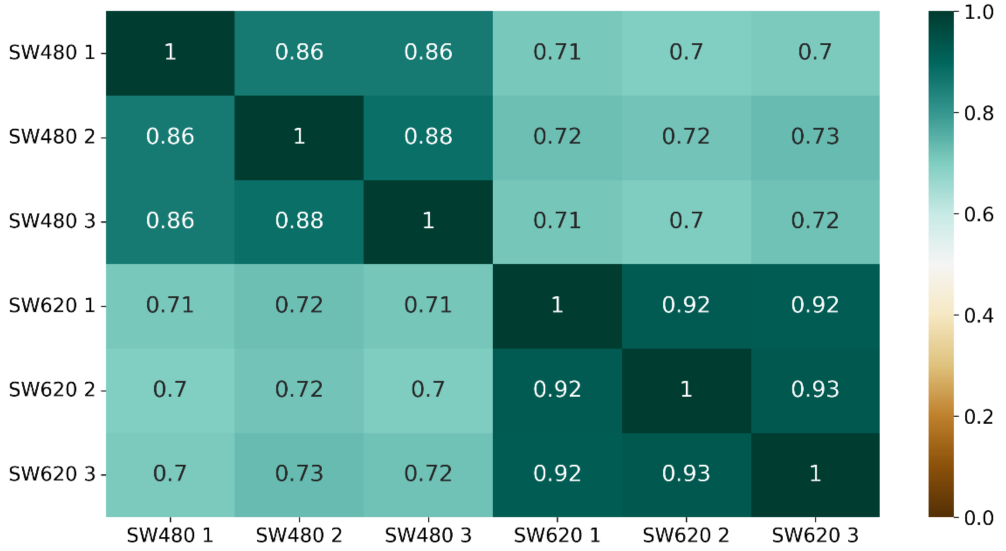

Fig. S9. **The heat map of Pearson correlation coefficients of proteoform intensities between technical replicates of one cell line and between two cell lines.** The shared proteoforms across the six different conditions were used for the analyses. The log2 transformation was used for proteoform intensity before calculating the Pearson correlation coefficients.

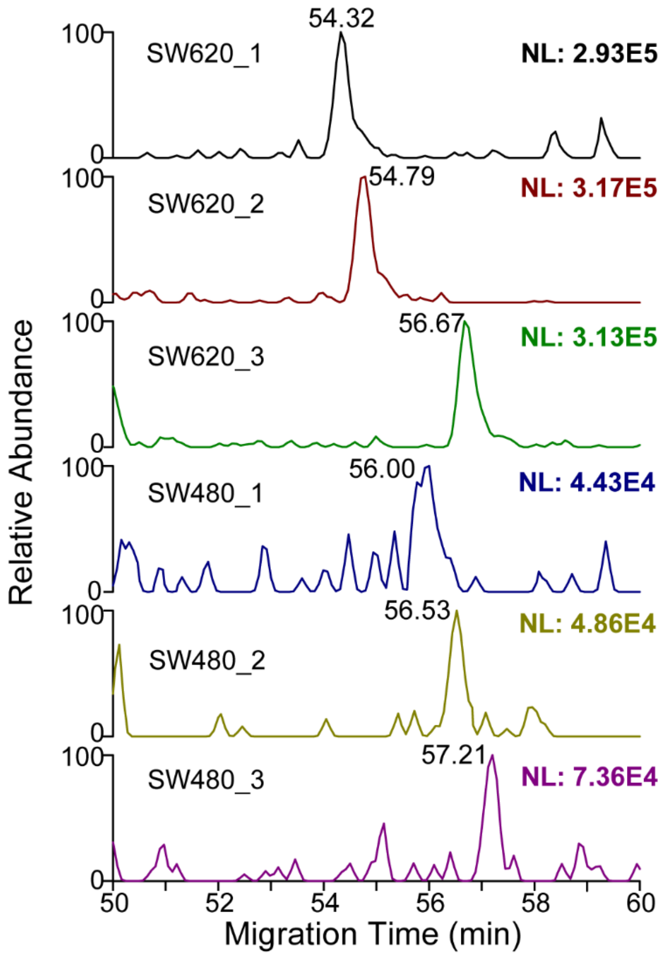

Fig. S10. **Extracted ion electropherograms (EIEs) of the NPM1 phosphorylated proteoform in Table S2 from SW480 and SW620 cells after triplicate CZE-MS/MS analyses.** The proteoform sequence is K.(C)[Carbamidomethylation]GSGPVHISGQHLVAVEEDAE(SE)[mass shift: 79.9682 Da, one phosphorylation]DEEEEDVKLLSISGKR.S. Precursor m/z corresponding to the identified charge state was extracted using 10 ppm mass tolerance and Gaussian smoothing (5 points). Migration time alignment was performed between the SW480 and SW620 data based on the accurate m/z and charge information.

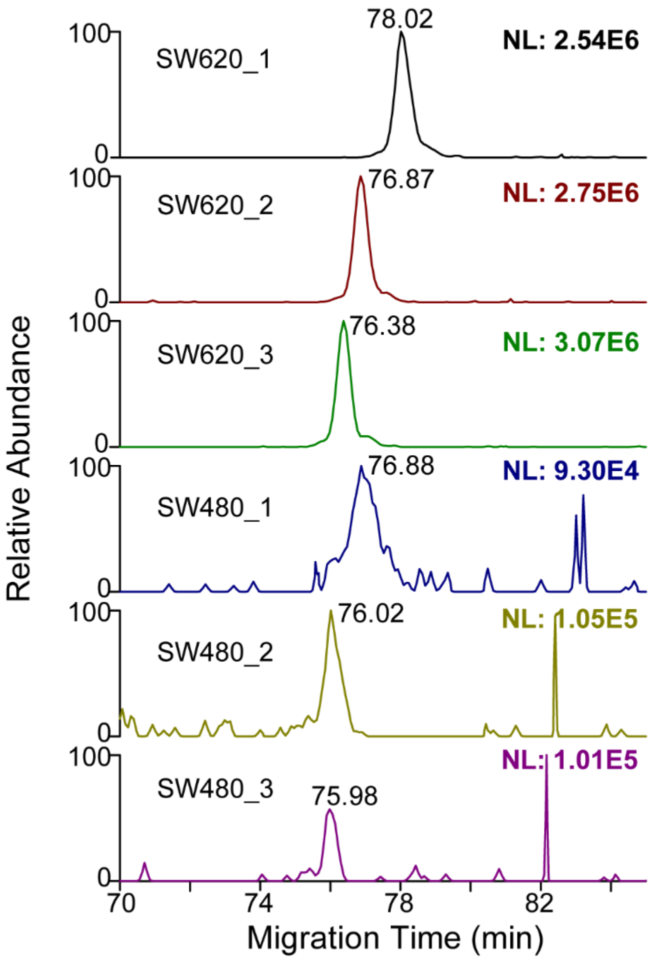

Fig. S11. Extracted ion electropherograms (EIEs) of the RALY phosphorylated proteoform in Table S2 (R.TRDDGDEEGLLTH(SEEELE)[mass shift: 79.9695 Da, one phosphorylation]HSQDTDADDGALQ) from SW480 and SW620 cells after triplicate CZE-MS/MS analyses. Precursor m/z corresponding to the identified charge state was extracted using 10 ppm mass tolerance and Gaussian smoothing (5 points). Migration time alignment was performed between the SW480 and SW620 data based on the accurate m/z and charge information.

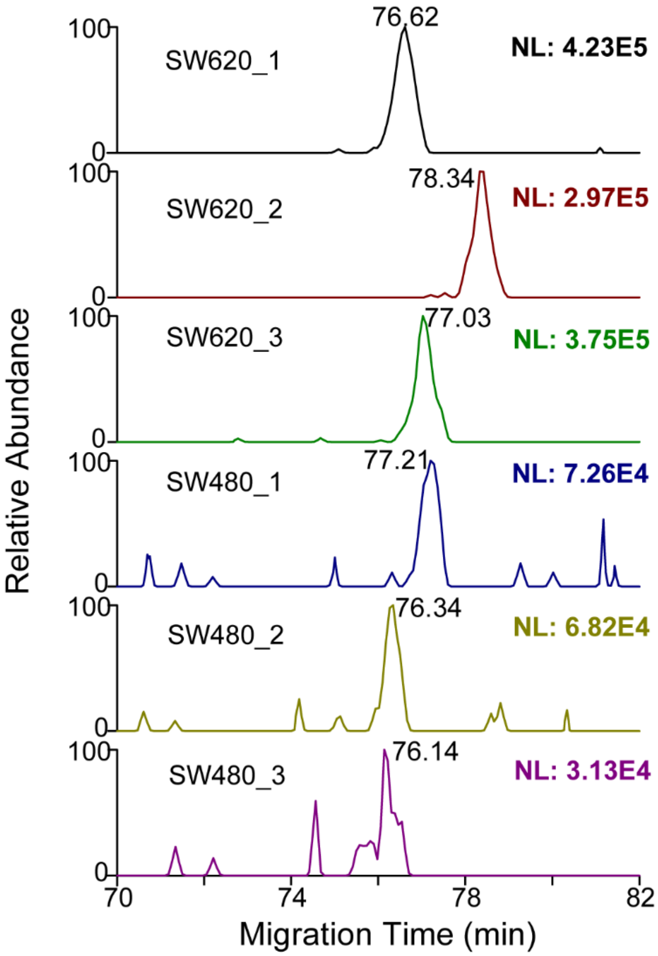

Fig. S12. **Extracted ion electropherograms (EIEs) of the HNRNPC phosphorylated proteoform in Table S2 (R.SAAEMYGSVTEH(PS)[mass shift: 79.9690 Da, one phosphorylation] PSPLLSSSFDLDYDFQRDYYDR.M) from SW480 and SW620 cells after triplicate CZE-MS/MS analyses.** Precursor m/z corresponding to the identified charge state was extracted using 10 ppm mass tolerance and Gaussian smoothing (5 points). Migration time alignment was performed between the SW480 and SW620 data based on the accurate m/z and charge information.

Table S1. **Summary of proteoform and protein identifications per sample in literature studies and this work.** The sample, strategy, instrument, MS run count, the number of proteoform identification, and the number of protein identification are listed.

Study	Sample/ starting protein material	Strategy	Mass Spectrometer	MS run count/sample	Proteoform count/sample	Protein count/sample	Reference
1	HeLa S3 cells<br><br>>4 mg of proteins	IEF-GELFrEE-RPLC-MS/MS	12T LTQ-FTICR	>360	3,000	1,043	Tran et al. (ref 18)
2	H1299 cells<br><br>>4 mg of proteins	Subcellular Fractionation-IEF- GELFrEE-RPLC-MS/MS	Orbitrap Elite	811	5,000	1,220	Catherman et al. (ref 19)
3	DLD-1 cells<br><br>400 µg of proteins	GELFrEE-RPLC-MS/MS	21 T FT-ICR	40	3,238	684	Anderson et al. (ref 20)
4	*E.coli* cells<br><br>1 mg of proteins	SEC-RPLC-CZE-MS/MS	Q-Exactive HF	43	5,705	850	McCool et al. (ref 23)
5	22 samples: 21 human cell types and plasma<br><br>Protein amount: not mentioned	Subcellular Fractionation-IEX-GELFrEE-RPLC-MS/MS	Orbitrap Fusion Lumos, 21 Tesla FT-ICR, Q-Exactive HF, and Orbitrap Elite	4-367<br>Mean: 70	300-9,991<br>Mean: 2,582	79-1,065<br>Mean: 417	Melani et al. (ref 21)
6	SW480 and SW620 cells<br><br>500 µg of proteins per cell line	SEC-CZE-MS/MS	Q-Exactive HF	SW480:<br>18 (6 SEC fractions×3 replicates)<br><br>SW620:<br>18 (6 SEC fractions×3 replicates)	SW480:<br>5,855<br><br>SW620:<br>6,273<br><br>Mean:<br>6,064	SW480:<br>1,113<br><br>SW620:<br>1,292<br><br>Mean:<br>1,203	This work
7	SW480 and SW620 cells<br><br>~ 3 mg of proteins per cell line	CZE-MS/MS, SEC-CZE-MS/MS, RPLC-CZE-MS/MS, SEC-RPLC-CZE-MS/MS	Q-Exactive HF	SW480:<br>~200<br>SW620:<br>~200	SW480:<br>17,316<br>SW620:<br>14,504<br><br>Mean: 15,910	SW480:<br>1,872<br>SW620:<br>1,884<br><br>Mean: 1,878	This work

Table S2. **Summary of the phosphorylated proteoforms with differential expression between SW480 and SW620 cells.** The gene name, proteoform sequence with PTM annotation, and proteoform intensity ratio (log2) between SW480 and SW620 are listed.

Gene	Log2(proteoform intensity ratio, SW480/ SW620)	Proteoform Sequence
DAP	1.53	R.IVQKHPHTGDTKEEKDKDDQEWES(PSPPKPTV)[mass shift: 79.9696 Da, one phosphorylation]FISGVIARGDKDFPPAAAQVAHQKPHASMDKHPSPR.T
DAP	-1.49	R.IVQKHPHTGDTKEEKDKDDQEWES(PS)[mass shift: 79.9692 Da, one phosphorylation]PPKPTVFISGVIAR.G
HDGF	3.45	R.AGDLLED(SPK)[mass shift: 79.9689 Da, one phosphorylation] RPKEAENPEGEEKEAATLEVERPLPMEVEKNSTPSEPGSGRGPPQEEE EEEDEEEEATKEDAEAPGIRDHESL.
NPM1	-2.67	K.(C)[Carbamidomethylation]GSGPVHISGQHLVAVEEDAE (SE)[mass shift: 79.9682 Da, one phosphorylation]DEEEEDVKLLSISGKR.S
RALY	-5.52	R.TRDDGDEEGLLTH(SEEELE)[mass shift: 79.9695 Da, one phosphorylation]HSQDTDADDGALQ.
HIST1H1 B	2.42	M.(S)[Acetyl]ETAPAETATPA(PVEKS)[mass shift: 79.9702 Da, one phosphorylation]PAKKKATK.K
HMGN1	2.01	K.QAEVANQETKEDLPAEN(GETKTEESPAS)[mass shift: 159.9318 Da, two phosphorylation sites]DEAGEKEAKSD.
HNRNPC	-3.09	R.SAAEMYGSVTEH(PS)[mass shift: 79.9690 Da, one phosphorylation]PSPLLSSSFDLDYDFQRDYYDR.M

Caption for Suppl. Excel_seq1 containing the lists of identified proteoforms

The supplementary excel file contains lists of identified proteoforms from SW480 and SW620 cells using CZE-MS/MS-based strategies, including lists of proteoforms from two cell lines, a list of proteoforms from only SW480 cell line, a list of proteoforms from only SW620 cell line, lists of identified proteoforms related to four well-known CRC-related pathways (WNT/β-catenin Signaling, mTOR Signaling, ERK/MAPK Signaling, PI3K/AKT Signaling pathways), lists of differentially expressed proteoforms between SW480 and SW620 cells, and lists of proteoforms containing SAAVs.
